# Impact of Pretransplant Hepatic Encephalopathy on Liver Posttransplantation Outcomes

**DOI:** 10.1155/2013/952828

**Published:** 2013-11-13

**Authors:** Lewis W. Teperman

**Affiliations:** Division of Transplant Director, Mary Lea Johnson Richards Organ Transplantation Center, New York University Langone Medical Center, Rivergate 3, 403 E 34th Street, New York, NY 10016, USA

## Abstract

Patients with cirrhosis commonly experience hepatic encephalopathy (HE), a condition associated with alterations in behavior, cognitive function, consciousness, and neuromuscular function of varying severity. HE occurring before liver transplant can have a substantial negative impact on posttransplant outcomes, and preoperative history of HE may be a predictor of posttransplant neurologic complications. Even with resolution of previous episodes of overt or minimal HE, some patients continue to experience cognitive deficits after transplant. Because HE is one of the most frequent pretransplant complications, improving patient HE status before transplant may improve outcomes. Current pharmacologic therapies for HE, whether for the treatment of minimal or overt HE or for prevention of HE relapse, are primarily directed at reducing cerebral exposure to systemic levels of gut-derived toxins (e.g., ammonia). The current mainstays of HE therapy are nonabsorbable disaccharides and antibiotics. The various impacts of adverse effects (such as diarrhea, abdominal distention, and dehydration) on patient's health and nutritional status should be taken into consideration when deciding the most appropriate HE management strategy in patients awaiting liver transplant. This paper reviews the potential consequences of pretransplant HE on posttransplant outcomes and therapeutic strategies for the pretransplant management of HE.

## 1. Introduction

 Cirrhosis of the liver—the only cure for which is liver transplant—is associated with several serious complications, including ascites, spontaneous bacterial peritonitis, variceal bleeding, and hepatic encephalopathy (HE) [1]. Guidelines established by the American Association for the Study of Liver Diseases currently recommend referring patients with cirrhosis for liver transplant when their model for end-stage liver disease (MELD) score is ≥10 and their Child-Turcotte-Pugh (CTP) score is ≥7 or when they experience their first major complication (e.g., HE, ascites, or variceal bleeding) [2]. However, the current United Network for Organ Sharing allocation system only uses the MELD score for prioritizing adults for liver transplant [3]. The MELD scoring system evaluates a patient's short-term prognosis based on 3 common laboratory test results: serum bilirubin, international normalized ratio, and serum creatinine levels. However, this scoring system does not take into account several serious complications of cirrhosis, such as HE, when prioritizing patients for liver transplant [4]. This may have negative ramifications for patient care, as the development of HE may be associated with substantial morbidity, mortality, and cost.

 HE imposes a significant burden on patients, their families, and health care resources [5, 6]. HE is characterized by alterations in behavior, cognitive abilities, consciousness, and neuromuscular function [7]. It negatively affects patient quality of life (QOL), and patients may be unable to drive, work, or adequately care for themselves because of its effects [8–11]. Patients may be less compliant with all prescribed medications, and hospitalizations related to HE may increase patient exposure to opportunistic infections and be associated with substantial costs. Furthermore, HE may be an independent predictor of mortality in patients with chronic liver disease [12]. HE occurring before liver transplant can also have a substantial negative impact on posttransplant outcomes [13–22]. This paper will review the potential consequences of pretransplant HE on posttransplant outcomes and therapeutic strategies for the pretransplant management of HE.

## 2. Consequences of Pretransplant HE on Posttransplant Outcomes

 HE can be classified into 3 types based on the hepatic abnormality observed [7]. HE may occur in patients with acute liver failure (type A HE), in patients with portosystemic shunting but no intrinsic hepatocellular disease (type B HE), or, as in the majority of HE cases, in patients with cirrhosis and cirrhosis-related portosystemic shunting (type C HE). Because HE is a progressive neuropsychiatric condition, HE may be graded or scored based on the severity of the clinical manifestations, which may range from subtle neurologic abnormalities in mild cases to coma in severe cases. Minimal HE (sometimes referred to as covert HE), which may occur in nearly 70% of patients with cirrhosis [23], is not associated with any clinical signs of brain dysfunction [7], but patients experience cognitive abnormalities that can lead to QOL impairment [8, 24, 25]. Unlike minimal HE, overt HE can manifest as a wide spectrum of symptoms that can be observed clinically, including those related to motor and neuropsychologic functions. Overt HE has been shown to occur in nearly half of patients with cirrhosis [26] and, as with minimal HE, also has a substantial negative impact on QOL [7–10].

 Although research is ongoing, the pathogenesis of HE is believed to primarily involve the exposure of the brain to elevated neurotoxin levels, particularly ammonia and other gut-derived toxins, leading to cellular morphologic changes (e.g., astrocyte swelling) and the development of a variety of neurochemical, neurotransmitter, and neuroinflammatory changes [27, 28]. Many factors that can precipitate HE (e.g., hypokalemia, infection, and gastrointestinal bleeding) serve to increase the production of ammonia or other gut-derived toxins or to reduce toxin metabolism by the liver (e.g., dehydration, anemia) [29]. Although the exact pathophysiology of HE is unclear, data are accumulating to suggest that the cascade of neuropsychiatric and neuromuscular sequelae of HE may have a longer term or more permanent negative impact on patients with chronic liver disease than originally suspected.

## 3. Neurologic Complications after Transplant

 Neurologic complications are common following liver transplant and may include alterations in mental status, seizures, and focal motor deficits [30]. The majority (≥75%) of these complications are observed within the first month after liver transplant, suggesting a possible relationship between preoperative status and liver transplant rather than the effect of immunosuppression [13, 19, 31]. However, neurologic complications may be observed in the long term, even 1 year after transplant [19, 32]. Of the neurologic complications, encephalopathy is most commonly observed, although the reported incidence has varied widely from 12% to 84% of patients at some point postoperatively [19, 20, 31, 33–36]. A variety of factors may cause neurologic complications, including encephalopathy, infection (e.g., sepsis), perioperative complications, persistence of major portosystemic shunts, and immunosuppressant-associated toxicity. Neurologic complications have also been associated with a greater risk of patient mortality [13, 37]. Thus, encephalopathy can be seen as a neurologic complication in itself and as a potential cause of neurologic complications.

### 3.1. Impact of Pretransplant Overt HE on Neurologic Complications after Transplant

 Preoperative history of HE is a significant predictor of posttransplant neurologic complications. In a prospective analysis of 84 patients with chronic liver disease who had undergone a liver transplant, the presence of an abnormal neurologic exam suggestive of HE before transplant was an independent risk factor for developing in-hospital central nervous system complications after transplant (*P* = 0.007) [13]. In a retrospective study of 101 patients who had undergone a liver transplant, a history of HE was strongly associated with neurologic complications after transplant (univariate odds ratio (OR), 2.6; 95% confidence interval (CI), 1.1–6.4; *P* = 0.03). Furthermore, using a multivariate analysis, HE in the immediate preoperative period was associated with posttransplant neurologic complications (adjusted OR, 10.7; 95% CI, 3.8–29.9; *P* < 0.013) [20].

**Figure 1:**
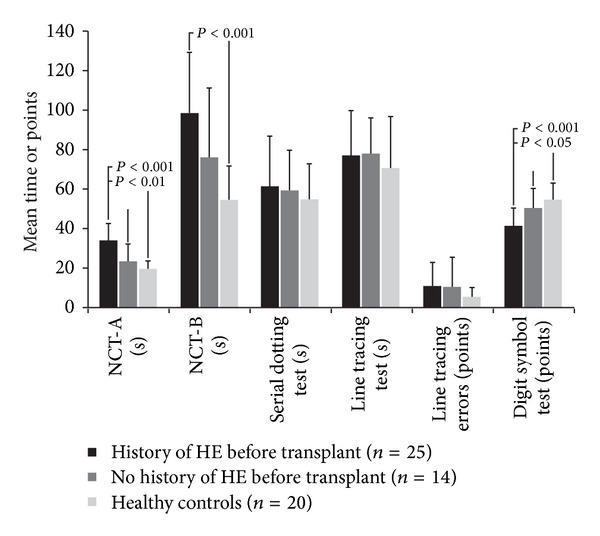
Psychometric HE score results for patients with a history of HE before transplant compared with patients with no history of HE before transplant and age-matched healthy individuals. HE, hepatic encephalopathy; NCT-A, number connection test A; NCT-B, number connection test B. Adapted with permission from Sotil et al., Copyright © 2009 American Association for the Study of Liver Diseases [21].

 Data continue to accumulate suggesting that even with resolution of prior episodes of overt HE, patients may continue to have cognitive deficits after transplant. Patients who had undergone a liver transplant, on average 17 to 19 months previously, received a battery of cognitive tests (e.g., psychometric hepatic encephalopathy score (PHES) and Repeatable Battery for the Assessment of Neuropsychological Status (RBANS; Pearson Education Inc., San Antonio, TX)) to determine if the presence of HE before liver transplant was associated with more substantial neurocognitive abnormalities within about 1.5 years after transplant [21]. Patients with a history of HE before transplant (*n* = 25) had significantly lower scores for 3 of 6 PHES domains compared with healthy individuals (*n* = 20) and for 2 of 6 PHES domains (attention domains) compared with patients without HE before transplant (*n* = 14; Figure 1) [21]. The investigators of this study did not determine the total PHES score because of a lack of available normative values. For RBANS, patients with a history of HE before transplant had significantly lower scores compared with healthy individuals in total RBANS score and 4 of the 5 RBANS subscores (*P* < 0.05). Although the total RBANS score, immediate memory, delayed memory, and attention subscores were lower for patients with HE before transplant than for patients without HE before transplant, no significant differences were observed [21].

 In cross-sectional (*n* = 226) and prospective assessments (*n* = 59) of patients with cirrhosis, patients who had experienced overt HE had greater cognitive dysfunction compared with patients without overt HE [22]. Patients who had an episode of overt HE had persistent impairment in cognitive function despite normalization of mental status on lactulose therapy, and the severity of impairment increased with the number of overt HE episodes. Thus, patients who experience overt HE may have persistent and cumulative deficits in working memory, response inhibitor, and learning that are chronic, cumulative, and not readily reversible (i.e., permanent) [22]. It is possible that these deficits may remain even after liver transplantation.

 Although not studied in patients who had eventually undergone a liver transplant, persistent cognitive impairment after overt HE was also supported by a study in 106 patients with cirrhosis currently without overt HE who were examined on 2 occasions within a 3-day period for the presence of mild cognitive impairment (PHES) [38]. Among 45 patients (42%) without a history of overt HE and 34 patients (32%) without a history of overt HE but with a current diagnosis of minimal HE, PHES results improved significantly from the first to the second exam (*P* = 0.04 and *P* = 0.016, resp.), suggesting a learning capacity for taking the tests involved in PHES [38]. However, there was no significant improvement in PHES results at the second exam for the 27 patients (25%) who had experienced at least 1 prior episode of overt HE, indicating a lack of learning capacity in patients with a history of overt HE [38]. Therefore, patients with a history of overt HE may have persistent cognitive impairment despite having a normal mental status and, in some cases, even in the presence of normal cognitive test results (PHES), which further supports the hypothesis that HE is not a fully reversible condition.

### 3.2. Impact of Pretransplant Minimal HE on Neurologic Complications after Transplant

 Evidence also exists for the posttransplant persistence of cognitive dysfunction or radiologic abnormalities in patients exhibiting minimal HE before transplant [14–18]. In a small prospective study, 14 patients with minimal HE underwent liver transplant and were assessed for visuomotor function (average time of assessment, 21 months after transplant) [14]. Improvement of visuomotor and visuoconstructive skills (e.g., trail making tests, reconstruction of drawing, or picture) was observed in some patients after transplant, but worsening was observed in others. Of note, no significant improvement in posttransplant visuomotor and visuoconstructive performance was noted compared with pretreatment performance, with 50% of patients showing deterioration in performance. In addition, mean posttransplant results for the 14 patients with minimal HE were significantly lower than for 22 age-matched healthy individuals (*P* = 0.04) [14].

In another study of patients with minimal HE before transplant (*n* = 23), most assessed cognitive functions improved at 6 months after transplant, with some cognitive functions improving only 18 months after transplant (e.g., verbal short-term memory) [39].

### 3.3. Mechanisms by Which HE Impacts Neurologic Complications after Transplant

**Figure 2:**
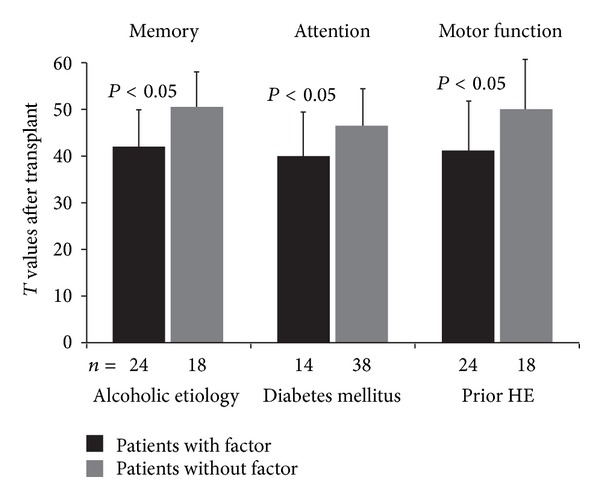
Posttransplant cognitive function (*T* values) in patients with or without risk factors that were determined to be associated with cognitive impairment (i.e., cirrhosis of alcoholic etiology, diabetes mellitus, and prior HE). *T* values were calculated using the following formula: *T* = 50 + 10 ([raw test value − mean test value]/SD of normal population). Impairment was defined as *T* ≤ 40. HE, hepatic encephalopathy; SD, standard deviation. Adapted with permission from Garcia-Martinez et al., Copyright © 2011 American Association for the Study of Liver Diseases [42].

 Data support the hypothesis that patients with a history of HE before transplant can have more pronounced cognitive dysfunction after transplant than patients without a history of HE. However, results are confounded by some studies that suggest almost complete normalization of radiologic findings after transplant, including gradual normalization of glutamine/glutamate and choline signals in a majority of patients as measured by magnetic resonance spectroscopy [40, 41]. In addition, the mechanisms by which more pronounced posttransplant cognitive impairment occurs in patients with pretransplant histories of HE are unclear, especially the lack of clearly defined variables that may play a role in short-term or long-term cognitive dysfunction. In a prospective study of 52 patients with cirrhosis who had a liver transplant (54% with minimal HE and 0% with overt HE), cognitive function significantly improved from pretransplant values for memory, attention, executive function, motor function, and visuospatial domains of the battery of tests administered (*P* < 0.05). However, 13% of patients still had global cognitive impairment 6 to 12 months after transplant [42]. In addition, after liver transplant, cognitive function in patients with cirrhosis of alcoholic etiology, diabetes mellitus, and prior HE was more severely impaired compared with patients without these factors. Posttransplant patients with alcohol-induced cirrhosis had memory decline, patients with diabetes mellitus exhibited attention impairment, and patients with histories of HE had impaired motor function (Figure 2) [42].

A multivariate analysis indicated that prior HE, diabetes mellitus, and cirrhosis of alcoholic etiology were considered risk factors for poor cognitive function that persists after transplant [42]. More research is necessary to gain a clearer understanding of the key demographics and disease characteristics that are involved to better identify patient subpopulations that are at the greatest risk for posttransplant neurologic complications.

## 4. Treatment of HE in the Pretransplant Setting

The development of minimal or overt HE before liver transplant may affect posttransplant outcomes, and HE is a probable risk factor for neuropsychiatric symptoms after transplantation. Therefore, although the cause of neuropsychiatric symptoms following a liver transplant is likely multifactorial, improving patient HE status before transplant may improve posttransplant outcomes. Current pharmacologic therapies for HE are primarily directed at reducing the systemic levels of ammonia and other toxins produced in the gastrointestinal tract, thereby reducing cerebral exposure. Patients may be treated for minimal HE, overt HE, or the prevention of HE relapse. However, most patients with minimal HE do not currently receive treatment outside the context of clinical trials. In all of these cases, the mainstays of therapy are nonabsorbable disaccharides and antibiotics.

### 4.1. Nonabsorbable Disaccharides

Nonabsorbable disaccharides, such as lactulose and lactitol (not available in the United States), are metabolized in the colon by intestinal bacteria, resulting in a reduction in colonic pH. The acidic environment promotes uptake of ammonia by colonic bacteria, facilitates diffusion of ammonia from the blood into the intestine, and may reduce the survival of urease-producing bacteria. Nonabsorbable disaccharides also increase the osmotic pressure of the intestinal lumen, which induces catharsis and elimination of potential sources of gut-derived toxins from the body.

A 2004 meta-analysis of 22 randomized studies was conducted to evaluate the efficacy of nonabsorbable disaccharides compared with no treatment, placebo, or antibiotics in patients with acute, chronic, or minimal HE [43]. Nonabsorbable disaccharides appeared to improve HE (i.e., reduced the risk of no improvement) when compared with no intervention or placebo (*P* = 0.002). However, when studies of poor methodologic quality were removed from the analysis, no significant effect was observed in the few high-quality trials that had been conducted. In addition, nonabsorbable disaccharides had no significant effect on mortality compared with no treatment or placebo intervention. The authors concluded that there was insufficient evidence to support or contest the use of lactulose or lactitol for the treatment of HE [43].

A 2011 meta-analysis of 5 studies specifically evaluated nonabsorbable disaccharides for the treatment of minimal HE and concluded that compared with placebo these agents significantly improved minimal HE (i.e., reduced the risk of no improvement) (*P* < 0.0001) [44]. Another 2011 meta-analysis of 9 studies evaluating lactulose for the treatment of minimal HE confirmed that lactulose prevented the progression to overt HE, compared with either placebo or no intervention. However, no significant difference in mortality was observed [45].

Subsequent to this analysis, an open-label randomized study concluded lactulose to be effective for the primary prophylaxis of overt HE in patients with cirrhosis [46]. Twenty (19%) of 105 patients followed for 12 months developed an episode of overt HE, six (11%) in the lactulose group and 14 (28%) in the nonlactulose treated group (*P* = 0.02) [46]. However, consistent with other studies, no significant difference in mortality was observed (*P* = 0.16).

In the 2004 meta-analysis, nonabsorbable disaccharides were significantly less effective than antibiotics in improving HE (i.e., they were associated with a higher risk of no HE improvement; *P* = 0.03) and did not have a significantly different impact on mortality [43]. Patients with HE who received nonabsorbable disaccharides also had higher blood ammonia levels after treatment compared with patients who received antibiotics [43]. However, a separate meta-analysis reported similar efficacy between antibiotics and nonabsorbable disaccharides in improving HE [47].

**Figure 3:**
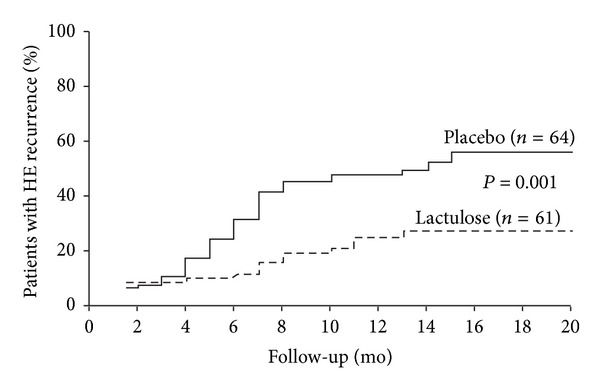
Probability of patients with cirrhosis developing HE recurrence during daily treatment with lactulose or placebo. HE, hepatic encephalopathy. Reprinted from Sharma et al., Copyright © 2009, with permission from W. B. Saunders Co. [49].

In addition, a few studies have evaluated nonabsorbable disaccharides for the prevention of HE recurrence (i.e., secondary prophylaxis) [48–51]. In 1 randomized, and unblinded, placebo-controlled study, 140 patients with cirrhosis who had recovered from a previous HE episode were randomly assigned within 1 week of recovery to receive either lactulose 30 to 60 mL/d (*n* = 70) or placebo (*n* = 70) [49]. Thirteen patients (9%) were lost to follow-up; 61 patients in the lactulose group and 64 patients in the placebo group were followed for a median of 14 months (range, 1–20 months). Lactulose significantly reduced the percentage of patients who experienced overt HE recurrence compared with placebo (20% versus 47%, resp.; *P* = 0.001; Figure 3) [49]. However, no significant difference in the median time of HE recurrence was observed (7.5 months (range, 1–13 months) versus 6.0 months (range, 2–15 months), resp.). In addition, no significant differences between the 2 groups were reported in admissions to the liver intensive care unit for conditions other than HE or deaths during the study [49].

 Adverse events (AEs) associated with nonabsorbable disaccharides are commonly gastrointestinal-related and include abdominal pain, diarrhea, flatulence, and nausea [43]. Diarrhea can also lead to secondary complications, including dehydration, hypokalemia, and hypernatremia [52]. Anorexia and vomiting have also been reported as AEs occurring with use of nonabsorbable disaccharides (rate of 2% for each) [53]. One additional concern with lactulose is that administration can cause abdominal distention, which may result in technical difficulties during liver transplant [54, 55]. These gastrointestinal AEs in patients awaiting liver transplant may directly impact patient nutritional status. Nutritional status before liver transplant has also been shown to correlate with posttransplant survival [56] and is independently associated with the number of infection episodes after transplant [57]. Malnutrition, assessed by a subjective global nutritional assessment exam, was found to be an independent risk factor for the length of stay in the intensive care unit and the total number of days spent in the hospital after transplant [57]. Furthermore, alterations in specific laboratory measures (e.g., albumin, sodium, and potassium) [58–60], which may be negatively impacted by gastrointestinal AEs, have also been identified as risk factors for surgical complications in patients who received a liver transplant, and serum sodium levels are a prognostic factor for survival in patients awaiting liver transplant [61, 62]. Thus, gastrointestinal AEs may increase patient risk, particularly for patients who are prone to malnutrition because of various comorbid variables or conditions (e.g., dietary restrictions or gastroparesis) [63]. It is possible that administration of nonabsorbable disaccharides such as lactulose may exacerbate pretransplant nutritional deficits [64], thereby contributing to poor posttransplant outcomes.

### 4.2. Antibiotics

 Antibiotics are administered to reduce systemic levels of ammonia and other gut-derived toxins by targeting gastrointestinal bacteria. Because of the risk for systemic AEs and bacterial antibiotic resistance with systemic antibiotics, nonsystemic antibiotics are preferred agents. Rifaximin is a nonsystemic gut-selective antibiotic and more than 20 studies have evaluated rifaximin for the treatment of overt HE (see review by Lawrence and Klee) [65] or minimal HE [66–68]. In 2010, rifaximin was approved by the US Food and Drug Administration for the maintenance of overt HE remission in adults [69].

**Figure 4:**
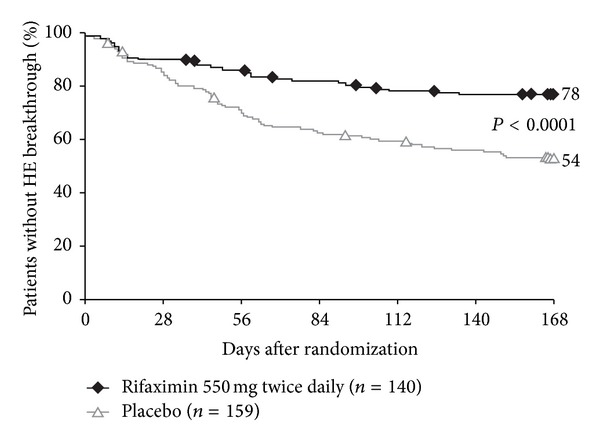
Kaplan-Meier estimate of time to first breakthrough HE episode (primary endpoint) in patients in remission for overt HE (Conn score, 0 or 1). HE breakthrough was defined as an increase in Conn score to ≥2 or, if baseline Conn = 0, a 1-unit increase each in Conn score and asterixis grade. Symbols indicate censored patients. HE, hepatic encephalopathy. Reprinted from Bass et al, Copyright © 2010, with permission from Massachusetts Medical Society [70].

 In a randomized, double-blind, phase 3 trial of rifaximin for the maintenance of HE remission, patients in remission from HE were treated with rifaximin 1100 mg/d (*n* = 140) or placebo (*n* = 159) for up to 6 months [70]. Concomitant lactulose administration was permitted during the study: 91% of patients in each group received concomitant lactulose. Only 22% of patients in the rifaximin group experienced a breakthrough HE episode compared with 46% of patients in the placebo group. Furthermore, rifaximin significantly reduced the risk of HE breakthrough by 58% compared with placebo during the 6 months of treatment (hazard ratio (HR), 0.42; 95% CI, 0.28–0.64; *P* < 0.001; Figure 4) [70]. Data indicated that the number needed to treat (NNT) was 4 (i.e., for every 4 patients treated with rifaximin for 6 months, 1 episode of breakthrough HE would be prevented). In addition, 14% of patients in the rifaximin group reported an HE-related hospitalization compared with 23% of patients in the placebo group [70]. Rifaximin significantly (*P* = 0.01) reduced the risk of HE-related hospitalizations by 50% compared with placebo (HR, 0.50; 95% CI, 0.29–0.87) [70]. Data indicated that the NNT was 9 (i.e., for every 9 patients treated with rifaximin for 6 months, 1 episode of HE-related hospitalization would be prevented).

Health-related QOL in the phase 3 trial was assessed using the Chronic Liver Disease Questionnaire (CLDQ), which was administered every 4 weeks, and the time to HE breakthrough recorded [70, 71]. A significant (*P* = 0.0087 to 0.0436) improvement with rifaximin treatment was noted in the overall CLDQ scores and in each domain score compared with placebo treatment, and scores were significantly (*P* < 0.0001) lower in patients who experienced HE breakthrough compared with those who remained in remission [71].

 In the phase 3 trial, rifaximin was well tolerated, with a similar incidence of AEs reported in both groups [70]. The most common AEs with rifaximin and placebo were nausea (14.3% versus 13.2%), diarrhea (10.7% versus 13.2%), fatigue (12.1% versus 11.3%), and peripheral edema (15.0% versus 8.2%). Two cases of *Clostridium difficile* infection (CDI) were reported during the double-blind portion of the trial, both in the rifaximin group. The authors noted that these 2 patients had multiple risk factors for CDI, including repeated hospitalizations during which they received multiple courses of antibiotic therapy, advanced age, and pantoprazole use. Both patients continued to receive rifaximin therapy during successful treatment for the CDI [70].

The impact of long-term rifaximin therapy on gut flora, including a risk of bacterial antibiotic resistance, is largely unknown. However, the drug appeared to have a protective effect against infections within 90 days after transplant (*P* = 0.026) in patients treated with rifaximin for HE during liver transplant candidacy and was not associated with a higher risk of multidrug-resistant bacterial infections [72].

Conventional antibiotics, neomycin and metronidazole, are also administered for the treatment of HE. However, strong clinical data supporting their efficacy in the treatment of HE are lacking. One randomized, double-blind study failed to demonstrate a benefit with neomycin (*n* = 20) compared with placebo (*n* = 19), with no significant differences observed for time to resolution of HE symptoms or for 5-day, 30-day, or 12-month mortality [73]. Two small, randomized, double-blind studies (*N* = 33 and *N* = 45) and 1 randomized, unblinded trial (*N* = 173) have suggested that neomycin and lactulose may have similar efficacy in the treatment of HE [74–76]. Two randomized studies (*N* = 35 and *N* = 49) have compared neomycin with rifaximin and suggested that they have similar efficacy in the treatment of HE, although in one of the studies, patients who received rifaximin showed improvements sooner than those who received neomycin (3 versus 5 days, resp.) [77, 78]. For metronidazole, 1 small study (*N* = 18) comparing metronidazole with neomycin suggested that both antibiotics improved mental state, reduced asterixis, and improved electroencephalogram measures [79].

However, the risk of AEs associated with neomycin and metronidazole may limit their use in patients with HE and suggest that they might not be an ideal pretransplant choice of treatment. Intestinal malabsorption and diarrhea have been observed with neomycin therapy [80] and thus could impact pretransplant nutritional status of the patients. Although neomycin is poorly absorbed, prolonged administration may result in cumulative systemic concentrations sufficient to increase the risk of serious AEs, such as ototoxicity and nephrotoxicity [80]. Because the MELD scoring system includes measures of renal dysfunction and neomycin may cause renal damage, neomycin may not be an ideal choice for patients, particularly those with high MELD scores. Metronidazole has been associated with peripheral neurotoxicity and requires dosing adjustments in patients with severe liver disease because of impaired drug clearance [80]. Metronidazole is a systemic antibiotic frequently administered for the treatment of CDI, and the potential risk of *C. difficile *resistance to metronidazole warrants judicious use of this agent [81].

Compared with nonabsorbable disaccharides, neomycin, and metronidazole, rifaximin exhibits a more favorable safety and tolerability profile [47, 65, 82]. Rifaximin has not been associated with gastrointestinal AEs such as diarrhea or nausea in clinical studies and would be unlikely to increase the risk of dehydration, weight loss, abdominal distention, malnutrition, or intestinal malabsorption in patients awaiting transplant, thereby minimizing the possible negative consequences of HE therapy on patient nutritional status.

A 2012 meta-analysis, incorporating data from 12 randomized controlled active comparator trials for the treatment of patients with HE, assessed the efficacy and psychometric outcomes of rifaximin compared with other oral therapies (including disaccharides and other antibiotics). The analysis showed that rifaximin exhibited comparable efficacy to other oral agents. However, more favorable effects were observed with rifaximin with regard to psychometric parameters and serum ammonia levels. A tolerability analysis, which included HE prevention trial data, indicated that rifaximin was also associated with fewer adverse effects [82].

## 5. Summary

 HE is a common complication of cirrhosis that substantially affects patient morbidity and mortality. Furthermore, HE can have a detrimental impact on posttransplant outcomes, including patient survival. Data continue to emerge demonstrating the potential persistence of cognitive deficits associated with HE, even after liver transplant. Therefore, prevention of HE in patients with cirrhosis may improve pretransplant health status and thus improve posttransplant outcomes. Commonly prescribed therapies include nonabsorbable disaccharides (e.g., lactulose) and nonsystemic antibiotics (e.g., rifaximin), and their various risks and benefits should be taken into consideration when deciding the most appropriate HE management algorithm in patients awaiting liver transplant. Further studies to evaluate currently available therapies in preventing HE and improving posttransplant outcomes are warranted.
